# Increased assessment of HER2 in metastatic gastroesophageal cancer patients: a nationwide population-based cohort study

**DOI:** 10.1007/s10120-020-01039-7

**Published:** 2020-01-11

**Authors:** Willemieke P. M. Dijksterhuis, Rob H. A. Verhoeven, Sybren L. Meijer, Marije Slingerland, Nadia Haj Mohammad, Judith de Vos-Geelen, Laurens V. Beerepoot, Theo van Voorthuizen, Geert-Jan Creemers, Martijn G. H. van Oijen, Hanneke W. M. van Laarhoven

**Affiliations:** 1 Department of Medical Oncology, Cancer Center Amsterdam, Amsterdam UMC, University of Amsterdam, Meibergdreef 9, 1105 AZ Amsterdam, The Netherlands; 2 Department of Research and Development, Netherlands Comprehensive Cancer Organisation (IKNL), Utrecht, The Netherlands; 3 Department of Pathology, Cancer Center Amsterdam, Amsterdam UMC, University of Amsterdam, Amsterdam, The Netherlands; 4 Department of Medical Oncology, Leiden University Medical Center, Leiden, The Netherlands; 5 Department of Medical Oncology, University Medical Center Utrecht, Utrecht University, Utrecht, The Netherlands; 6 Department of Internal Medicine, Division of Medical Oncology, GROW-School for Oncology and Developmental Biology, Maastricht UMC+, Maastricht, The Netherlands; 7 Department of Medical Oncology, Elisabeth-TweeSteden Hospital, Tilburg, The Netherlands; 8 Department of Medical Oncology, Rijnstate Hospital, Arnhem, The Netherlands; 9 Department of Medical Oncology, Catharina Hospital, Eindhoven, The Netherlands

**Keywords:** Esophageal neoplasms, Gastric neoplasms, Adenocarcinoma, ErbB-2 receptor, Drug therapy, Trastuzumab

## Abstract

**Background:**

Addition of trastuzumab to first-line palliative chemotherapy in gastroesophageal cancer patients with HER2 overexpression has shown to improve survival. Real-world data on HER2 assessment and administration of trastuzumab are lacking. The aim of this study was to assess HER2 testing, trastuzumab administration, and overall survival (OS) in a nationwide cohort of metastatic gastroesophageal cancer patients.

**Methods:**

Data of patients with synchronous metastatic gastroesophageal adenocarcinoma diagnosed in 2010–2016 that received palliative systemic treatment (*n* = 2846) were collected from the Netherlands Cancer Registry and Dutch Pathology Registry. The ToGA trial criteria were used to determine HER2 overexpression. Proportions of HER2 tested patients were analyzed between hospital volume categories using Chi-square tests, and over time using trend analysis. OS was tested using the Kaplan Meier method with log rank test.

**Results:**

HER2 assessment increased annually, from 18% in 2010 to 88% in 2016 (*P* < 0.01). Median OS increased from 6.9 (2010–2013) to 7.9 months (2014–2016; *P* < 0.05). Between the hospitals, the proportion of tested patients varied between 29–100%, and was higher in high-volume hospitals (*P* < 0.01). Overall, 77% of the HER2 positive patients received trastuzumab. Median OS was higher in patients with positive (8.8 months) and negative (7.4 months) HER2 status, compared to non-tested patients (5.6 months; *P* < 0.05).

**Conclusion:**

Increased determination of HER2 and administration of trastuzumab have changed daily practice management of metastatic gastroesophageal cancer patients receiving palliative systemic therapy, and possibly contributed to their improved survival. Further increase in awareness of HER2 testing and trastuzumab administration may improve quality of care and patient outcomes.

**Electronic supplementary material:**

The online version of this article (10.1007/s10120-020-01039-7) contains supplementary material, which is available to authorized users.

## Introduction

Palliation by systemic therapy may improve quality as well as quantity of life in patients with metastatic gastroesophageal cancer [1-5]. In clinical trials, the addition of the targeted agent trastuzumab to cytotoxic therapy in metastatic gastroesophageal junction (GEJ) and gastric adenocarcinoma patients with overexpression of the human epidermal growth factor receptor 2 (HER2) has resulted in a median overall survival (OS) benefit of 2.8 months [6], and a positive impact on quality of life [7]. Trastuzumab has therefore become standard of care in HER2 positive tumors, and HER2 testing is strongly recommended in all patients with metastatic gastroesophageal adenocarcinoma eligible for HER2 targeted treatment [8-13].

HER2 testing and the administration of trastuzumab in gastroesophageal cancer might be underexposed within individual centers, because gastroesophageal cancer has a relatively low incidence in Western countries, and only 15–25% of the adenocarcinomas show HER2 overexpression [14-16]. In recent years, several studies have been published showing that gastroesophageal cancer patients treated in high-volume hospitals have better outcomes [17-25]. Patient volume can therefore be regarded as a proxy for quality of care, possibly due to multimodal expertise and a well-developed organization of care in high-volume hospitals [22, 26]. Moreover, although HER2 testing is routinely performed in breast cancer, HER2 expression in gastroesophageal cancer is more heterogenous as a reflection of the distinct biology of these tumors, and as a result, the interpretation of HER2 immunohistochemistry (IHC) patterns is more complicated [6, 27, 28].

Currently, data on HER2 testing, and the administration of trastuzumab in clinical practice are lacking. In this real-world study covering a nationwide cohort of synchronous metastatic gastroesophageal adenocarcinoma patients treated with systemic therapy, our aim was to explore the rate of HER2 testing, the administration of trastuzumab, interhospital variation, and survival in these patients.

## Methods

### Data collection

Patients with synchronous metastatic gastroesophageal adenocarcinoma (classified as C15 and C16 according to the International Classification of Diseases for Oncology [29]) treated with systemic therapy, and therefore eligible for HER2 targeted therapy, were selected from the nationwide Netherlands Cancer Registry (NCR). All patients diagnosed in 2010–2015 were identified, and a subset of patients diagnosed in 2016 because not all patients were registered in the NCR at the time of selection. Pathology reports of all confirmed cancer diagnoses in the Netherlands are archived in the nationwide network and registry of histo- and cytopathology (PALGA) [30]. Every pathology laboratory in the Netherlands is part of the PALGA network, and excerpts of all pathological reports are automatically transferred from the laboratories to the central databank of PALGA. Also, modifications in the excerpts or results of additional pathological tests, e.g., HER2 testing, are added to the central database automatically. Of included patients, information of HER2 testing was extracted from PALGA reports concerning histologic material with gastroesophageal origin.

Data on patient and tumor characteristics were extracted from the hospital’s medical records by trained data managers, and information on vital status from the Dutch population register (updated until 1 February 2019). OS was assessed from start of treatment until death or end of follow-up. Time to failure (TTF) was calculated from the start of treatment to the first progression that resulted in termination of first-line treatment, end of follow-up, or death within 90 days after the last hospital visit in case no progression was registered. Details on systemic treatment regimen and TTF were available in patients who were diagnosed in 2015 (*n* = 445) or in a subset of Dutch hospitals between 2010 and 2014 (*n* = 1107), due to logistic reasons. The subset of hospitals was selected as a representative sample of all Dutch hospitals in terms of patient volume, and hospital type and location, as described earlier [31].

### Patient selection

**Fig. 1:**
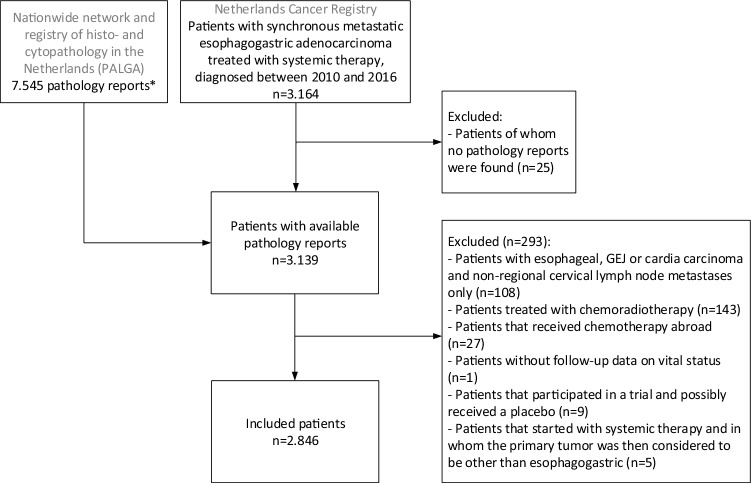
Flow chart of patient selection. Patients with an esophageal or gastroesophageal junction or cardia carcinoma with solely non-regional head and neck lymph node metastases were excluded, because they could have received definitive chemoradiotherapy with potential curative intent in case of involvement of only the supraclavicular lymph nodes. *Of the majority of the patients (*n* = 1990), more than one report was included. *GEJ* gastroesophageal junction

The NCR provided 3164 patients diagnosed with gastroesophageal adenocarcinoma and synchronous metastases diagnosed between 2010 and 2016 and treated with systemic therapy. The linkage of the NCR and the PALGA database identified 3139 patients, with a total of 7545 available pathology excerpts (Fig. 1).

### HER2 status

HER2 testing is usually initiated by the treating clinician in The Netherlands. A validated testing algorithm for HER2, based on the results of the ToGA trial [28], is suggested in international and national guidelines [8-10, 32-34]. The Dutch gastric cancer guideline recommends the use of validated HER2 antibodies for IHC and validated ISH tests, and a scoring system for the interpretation of these tests as described by Rüschoff et al. [27, 34] A gastroesophageal tumor can be considered HER2 positive when the result of the IHC staining pattern is 3+ , and negative when it is 0 or 1+ . In case of a equivocal IHC result (2+), additional testing using in situ hybridization (ISH) is indicated. The definition of HER2 ISH positivity is a HER2:chromosome 17 ratio of ≥ 2 [27]. Genomic testing techniques such as multiplex ligation-dependent probe amplification (MLPA) are used instead of ISH as well [12].

HER2 status was regarded as unknown if type and/or results of testing were not reported, because we could not verify if the HER2 criteria of the ToGA trial were used. In case of an equivocal IHC with an unknown ISH or MLPA result, HER2 status was also assumed unknown. If HER2 was tested multiple times, the last test result that was performed prior to or within 31 days after start of first-line systemic treatment was considered the definitive result, because this was expected to be decisive for the choice of systemic treatment. If HER2 testing was not mentioned in the reports, we assumed that it had not been performed.

### Hospital volume

Per hospital the volume of all gastroesophageal cancer patients (both adenocarcinoma and squamous cell carcinoma) that received systemic therapy in 2015–2016, regardless of tumor stage and the intent of treatment, was calculated. With the aim to reflect current practice, the volume of the two most recent years, was used. Hospitals were categorized into quartiles according to these volumes to compare the proportion of HER2 tested patients.

### Statistical analyses

Baseline characteristics and details on HER2 testing were displayed with counts and percentages, or medians and interquartile ranges (IQRs). Differences in the proportions of HER2 tested patients between the hospital volume categories were analyzed using Chi-square tests, and over time using the Cochran-Armitage test for trend. Factors possibly associated with HER2 testing were identified using logistic regression. Differences in survival were tested univariably with the log rank test using Kaplan Meier curves and through multivariable proportional hazards regression analyzes with adjustment for relevant patient and tumor characteristics. For survival analyzes, patients in whom HER2 was tested > 31 days after first-line systemic treatment were excluded to reduce immortal time bias. *P* values below 0.05 were considered statistically significant. Analyses were performed using SAS software (version 9.4, SAS institute, Cary, NC, USA).

## Results

### Patient characteristics

The majority of all 2846 included patients was male (76%), and median age was 64 (IQR, 56–71) years (Table 1). The primary tumor location was the esophagus in 41%, the non-cardia stomach in 40% and GEJ/cardia in 19%. More than half (54%) of the patients had an intestinal-type adenocarcinoma, followed by 27% with a diffuse, and 6% with an indeterminate type, based on the Lauren’s criteria [35]. In 13%, histological type was not specified. The majority of the tumors had a poor differentiation (53%).

**Table 1:** Baseline characteristics of included patients (*n* = 2846)

	All patients (*n* = 2846) no. (%)	Non-tested (*n* = 1322) no. (%)	HER2 tested (*n* = 1524) no. (%)	*P* value
Male	2152 (75.6%)	1023 (77.4%)	1129 (74.1%)	0.041a
Age (years) median (IQR)	64.0 (56.0, 71.0)	65.0 (58.0, 72.0)	63.0 (55.0, 69.0)	< 0.001^b^
< 50	963 (33.8%)	383 (29.0%)	580 (38.1%)	< 0.001^a^
50–64	1079 (37.9%)	510 (38.6%)	569 (37.3%)	
65–79	735 (25.8%)	386 (29.2%)	349 (22.9%)	
≥ 80	69 (2.4%)	43 (3.3%)	26 (1.7%)	
Comorbidities				< 0.001^a^
0	739 (26.0%)	285 (21.6%)	454 (29.8%)	
1	555 (19.5%)	245 (18.5%)	310 (20.3%)	
≥ 2	629 (22.1%)	278 (21.0%)	351 (23.0%)	
Unknown	923 (32.4%)	514 (38.9%)	409 (26.8%)	
Tumor location				< 0.001^a^
Esophageal	1159 (40.7%)	589 (44.6%)	570 (37.4%)	
Gastroesophageal junction/cardia	545 (19.1%)	219 (16.6%)	326 (21.4%)	
Stomach (non-cardia)	1142 (40.1%)	514 (38.9%)	628 (41.2%)	
Tumor histology				0.012^a^
Adenocarcinoma NOS	362 (12.7%)	160 (12.1%)	202 (13.3%)	
Intestinal-type adenocarcinoma	1540 (54.1%)	744 (56.3%)	796 (52.2%)	
Diffuse type adenocarcinoma	772 (27.1%)	327 (24.7%)	445 (29.2%)	
Indeterminate type adenocarcinoma	172 (6.0%)	91 (6.9%)	81 (5.3%)	
Tumor differentiation				< 0.001^a^
Well differentiated	37 (1.3%)	15 (1.1%)	22 (1.4%)	
Moderately differentiated	495 (17.4%)	221 (16.7%)	274 (18.0%)	
Poorly differentiated	1496 (52.6%)	648 (49.0%)	848 (55.6%)	
Unknown	818 (28.7%)	438 (33.1%)	380 (24.9%)	
Metastatic sites				< 0.001^a^
1	1571 (55.2%)	757 (57.3%)	814 (53.4%)	
≥ 2	1275 (44.8%)	565 (42.7%)	710 (46.6%)	
Year of diagnosis				< 0.001^a^
2010	414 (14.5%)	341 (25.8%)	73 (4.8%)	
2011	386 (13.6%)	265 (20.0%)	121 (7.9%)	
2012	423 (14.9%)	240 (18.2%)	183 (12.0%)	
2013	410 (14.4%)	172 (13.0%)	238 (15.6%)	
2014	451 (15.8%)	156 (11.8%)	295 (19.4%)	
2015	445 (15.6%)	109 (8.2%)	336 (22.0%)	
2016	317 (11.1%)	39 (3.0%)	278 (18.2%)	

Tumor histology and differentiation are based on the primary tumor. Diffuse type tumors were classified as poorly differentiated

*IQR* interquartile range, *NOS* not otherwise specified

^a^Chi square test

^b^Mann–Whitney *U* test

### HER2 testing

**Fig.2:**
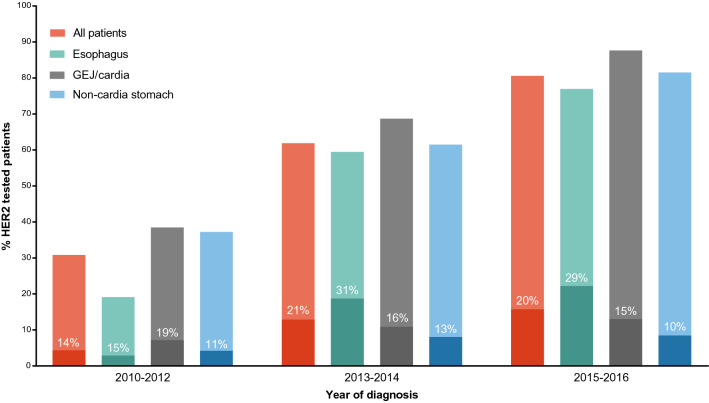
HER2 testing and overexpression stratified for primary tumor location. Proportion of HER2 tested patients over time. The percentages within the bars reflect the proportion of patients of whom the tumor showed HER2 overexpression. *GEJ* gastroesophageal junction

HER2 status was determined in 54% of the patients (*n* = 1524; Table 1). The proportion of tested patients increased over time (*P* < 0.001), from 18% in 2010, to 88% in 2016 (Fig. 2). This trend was seen in esophageal (11–89%), GEJ (24–93%) and gastric tumors (22–83%; all *P *< 0.001). HER2 tested patients were significantly younger, more often female, and had more frequently GEJ/cardia or stomach compared to esophageal tumors, and diffuse type adenocarcinomas than non-tested patients (Table 1).

HER2 was positive in 19% of 1524 tested patients, and negative in 68% (Supplementary Table 1). In 204 (13%) patients, HER2 status was unknown because detailed HER2 test results were not described. The number of HER2 positive tumors increased from 14% in 2010–2012 to 20% in 2015–2016 (Fig. 2). Overall, HER2 positivity was found in 28% of esophageal, 16% of GEJ/cardia, and 12% of gastric adenocarcinomas (*P* < 0.001).

### HER2 testing methods

Table 3 displays which diagnostic methods were used for the HER2 assessment, and all test results. IHC, ISH and MLPA were used in 88%, 49%, and 3% of the 1524 tested patients, respectively. Supplementary Table 2 displays which diagnostic methods were used for the HER2 assessment, and all test results of all performed tests. IHC, ISH and MLPA were used in 88%, 49%, and 3% of the 1524 tested patients, respectively, while testing methods were unknown in 13%. Of the patients in whom IHC was performed (*n* = 1328), scores of 0, 1+ , 2+ , 3+ were found in 38%, 23%, 24%, and 14%, respectively.

HER2 testing was performed more than once in 225 patients: in 194 patients, it was tested twice, in 30 patients three times, and in one patient four times. In 87% of the tested patients, HER2 was determined on solely the primary tumor, followed by metastasis only in 7%, and on both the primary tumor and metastasis in 6% of the patients. Testing methods were known in 1537/1764 tests, and in 398/1537 (26%) of these tests, ISH was used despite an IHC test result that would not necessarily require further testing (0, 1+ or 3+ ; Table 3).

### Hospital variation

**Fig. 3:**
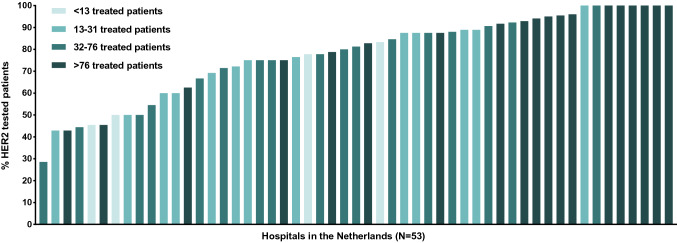
Hospital variation in HER2 testing in The Netherlands. Proportion of patients treated with systemic therapy for whom HER2 was assessed in 53 Dutch hospitals in 2015 and 2016. Each bar represents a hospital. Hospitals that treated less than six metastatic gastroesophageal adenocarcinoma patients with palliative systemic therapy in 2015 and 2016 were not displayed (*n* = 24)

The subdivision of hospitals resulted in volume categories of < 13, 13–31, 32–76 and > 76 patients treated with systemic therapy in 2015 and 2016. The proportion of HER2 tested patients differed between these volumes in patients diagnosed in 2015–2016 (*P* < 0.001), with the highest proportions of tested patients being found in the high-volume centers (88%; Table 2). Interhospital variation in HER2 tested was 29–100% (Fig. 3).

**Table 2:** HER2 testing by hospital volume of systemic treatment in 2015–2016

Hospital volume	Hospitals no.	Patients no.	HER2 tested patients no. (%)	*P* value
< 13 patients	17	72	49 (68.1%)	< 0.001
13–31 patients	19	157	119 (75.8%)	
32–76 patients	19	231	179 (77.5%)	
> 76 patients	19	302	267 (88.4%)	

Hospital volume-based differences in proportion of HER2 tested patients with metastatic gastroesophageal cancer diagnosed in 2015 and 2016. Hospitals are categorized in quartiles based on the hospital volume of all gastroesophageal cancer patients treated with systemic therapy in 2015 and 2016

### Factors contributing to probability of HER2 testing

Male sex, all but the highest hospital volumes, and death within 90 days after start of treatment were independently associated with lower probability of being tested for HER2 in patients diagnosed in 2015–2016, and GEJ/cardia tumors with a higher chance of testing to non-cardia gastric tumors (Table 3).

**Table 3:** Multivariable logistic regression analyses for chance of HER2 assessment for patients with metastatic gastroesophageal cancer treated with systemic therapy and diagnosed in 2015 and 2016 (*n* = 762)

		Univariable			Multivariable		
	Patients no	OR	95%CI OR	*P* value	OR	95%CI OR	*P* value
Sex							
Female	166	Ref			Ref		
Male	596	0.41	0.24–0.69	0.001	0.43	0.24–0.77	0.005
Age (years)							
< 50	235	Ref			Ref		
50–64	292	0.67	0.42–1.06	0.087	0.84	0.51–1.40	0.510
65–79	216	0.58	0.36–0.94	0.026	0.65	0.38–1.11	0.113
≥ 80	19	0.37	0.13–1.03	0.057	0.37	0.12–1.14	0.084
Performance status							
0 or 1	490	Ref			Ref		
≥ 2	49	0.78	0.42–1.48	0.453	0.99	0.50–1.97	0.981
Unknown	223	0.66	0.45–0.98	0.040	0.73	0.47–1.12	0.144
Number of comorbidities							
0	236	Ref					
1	208	0.61	0.37–1.00	0.048	0.62	0.36–1.06	0.081
≥ 2	256	0.54	0.34–0.87	0.011	0.66	0.39–1.11	0.119
Unknown	62	0.68	0.33–1.41	0.295	0.50	0.23–1.10	0.087
Primary tumor location							
Esophagus	356	0.76	0.51–1.13	0.170	0.80	0.51–1.24	0.315
GEJ/cardia	146	1.61	0.90–2.89	0.110	2.09	1.12–3.90	0.020
Stomach	260	Ref			Ref		
Year of diagnosis							
2015	445	Ref			Ref		
2016	317	2.31	1.55–3.45	< 0.001	2.54	1.67–3.88	< 0.001
Hospital volume							
< 13 patients	72	0.28	0.15–0.51	< 0.001	0.26	0.14–0.51	< 0.001
13–31 patients	157	0.41	0.25–0.68	< 0.001	0.37	0.22–0.64	< 0.001
32–76 patients	231	0.45	0.28–0.72	< 0.001	0.46	0.28–0.75	0.002
> 76 patients	302	Ref			Ref		
Deceased within 90 days after start systemic therapy	166	0.56	0.38–0.84	0.005	0.61	0.38–0.95	0.029

Multivariable logistic regression analyses for HER2 testing in patients diagnosed in 2015 and 2016

*OR* odds ratio, *CI* confidence interval, *GEJ* gastroesophageal junction

### Survival

OS was 7.3 (IQR, 3.5, 12.6) months in all 2846 patients, and increased from 6.9 (IQR, 3.5, 12.0) months in patients diagnosed in 2010–2013 (*n* = 1633) to 7.9 (IQR, 3.6, 13.6) months in 2014–2016 (*n* = 1213). OS was 6.2 (IQR, 2.9, 10.7) months in non-tested patients (*n* = 1322) versus 8.3 (IQR, 4.2, 14.4) months in HER2 tested patients (*n* = 1524).

**Fig. 4:**
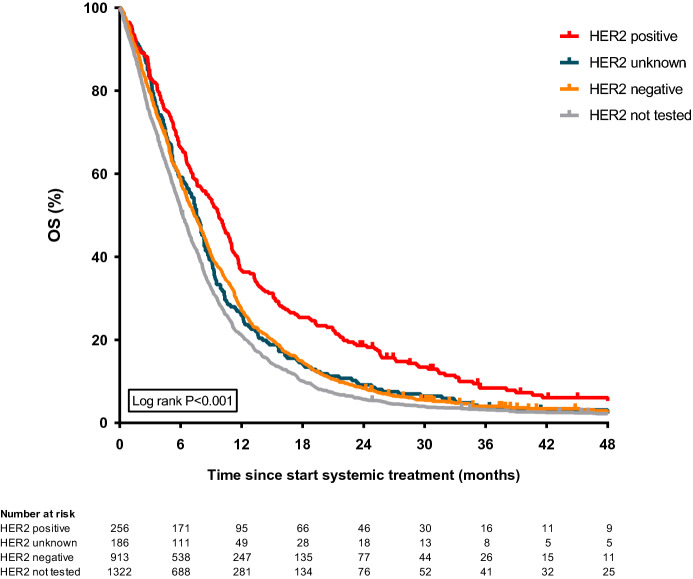
Kaplan Meier curve for overall survival in HER2 tested and non-tested patients. Overall survival in patients in whom HER2 overexpression was not determined (*N* = 1322), and in patients in whom HER2 was determined before or within a month after start of systemic treatment (*N* = 1355) categorized in negative (*N* = 913), positive (*N* = 256) and unknown (patients in whom test results were not specified; *N* = 186) HER2 status. Survival of patients in whom HER2 was determined after 31 days of start of systemic treatment (*N* = 169) is not displayed to prevent an immortal time bias. *OS* overall survival

In 1355 patients that were tested within 1 month after start of systemic treatment, median OS in patients with unknown, negative and positive HER2 status was 7.6, 7.4, and 9.8 months, respectively (Fig. 4). OS was significantly higher in HER2 positive and negative patients, compared to non-tested patients (HER2 negative: adjusted hazard ratio (HR) 0.81, 95% confidence interval (CI) 0.65–0.99; HER2 positive: HR 0.65, 95%CI 0.50–0.86; Table 4). Diffuse type tumors, ≥ 2 metastatic locations, and performance status ≥ 2 were independently associated with worse survival. Hospital volume categorized into 4 quartiles was not independently associated with OS, while HRs in the two lowest quartiles were 1.26 and 1.19, respectively. When hospital volumes were subdivided in low (below median) versus high (above median), patients treated in low-volume hospitals had a significantly worse survival (HR 1.19, 95%CI 1.00–1.41; *P* = 0.044).

**Table 4:** Multivariable Cox regression analyses overall survival in patients diagnosed between 2015 and 2016 (n = 735^a^)

	Patients no.		Median OS (months)	Univariable			Multivariable		
				HR	95%CI HR	*P* value	HR	95%CI HR	*P* value
Sex									
Female	161		6.1	Ref			Ref		
Male	574		7.6	0.94	0.79–1.13	0.520	0.98	0.80–1.19	0.804
Age (years)									
< 50	228		7.6	Ref			Ref		
50–64	282		7.3	1.02	0.85–1.22	0.833	1.05	0.87–1.27	0.641
65–79	207		6.5	1.00	0.82–1.22	0.988	1.04	0.84–1.28	0.754
≥ 80	18		5.8	1.33	0.82–2.16	0.245	1.20	0.72–1.99	0.477
Performance status									
0 or 1	459		7.8	Ref			Ref		
≥ 2	65		3.4	1.78	1.37–2.32	< 0.001	1.75	1.34–2.30	< 0.001
Unknown	211		6.0	1.20	1.02–1.42	0.033	1.19	1.00–1.42	0.047
Number of comorbidities									
0	225		7.6	Ref			Ref		
1	205		7.2	0.93	0.76–1.13	0.474	0.91	0.74–1.11	0.351
≥ 2	246		6.2	1.18	0.98–1.42	0.088	1.15	0.94–1.41	0.165
Unknown	59		8.9	0.89	0.66–1.19	0.427	0.83	0.61–1.13	0.231
Primary tumor location									
Esophagus	344		7.3	0.94	0.79–1.11	0.431	1.05	0.85–1.29	0.659
GEJ/cardia	143		7.8	0.85	0.69–1.05	0.138	0.96	0.76–1.21	0.703
Stomach	248		6.5	Ref			Ref		
Tumor histology									
Adenocarcinoma NOS	178		7.3	Ref			Ref		
Intestinal-type adenocarcinoma	350		8.0	1.00	0.83–1.21	0.992	0.95	0.77–1.19	0.671
Diffuse type adenocarcinoma	169		6.0	1.31	1.05–1.64	0.015	1.33	1.01–1.74	0.040
Indeterminate type adenocarcinoma	38		5.9	1.36	0.95–1.94	0.096	1.26	0.86–1.84	0.243
Number of metastatic locations									
1	389		7.7	Ref			Ref		
≥ 2	346		6.6	1.22	1.05–1.42	0.010	1.25	1.07–1.46	0.005
Year of diagnosis									
2015	429		6.7	Ref			Ref		
2016	306		7.7	0.88	0.76–1.03	0.107	0.95	0.79–1.14	0.580
Hospital volume^b^									
< 13 patients	70		5.2	1.28	0.97–1.67	0.081	1.26	0.95–1.67	0.104
13–31 patients	151		6.4	1.26	1.03–1.54	0.026	1.19	0.96–1.47	0.135
32–76 patients	220		7.0	1.11	0.93–1.33	0.247	1.04	0.86–1.25	0.669
> 76 patients	294		7.9	Ref			Ref		
HER2 status									
HER2 not tested	148		5.6	Ref			Ref		
HER2 tested, HER2 negative	392		7.4	0.77	0.64–0.94	0.010	0.81	0.66–1.00	0.040
HER2 tested, HER2 positive	118		8.8	0.62	0.48–0.80	< 0.001	0.66	0.49–0.85	0.002
HER2 tested, HER2 unknown	77		7.9	0.81	0.61–1.07	0.136	0.81	0.60–1.06	0.165

Multivariable Cox regression analyses for overall survival in patients diagnosed in 2015 and 2016

*OS* overall survival, *HR* hazard ratio, *CI* confidence interval, *GEJ* gastroesophageal junction, *NOS* not otherwise specified

^a^Patients in whom HER2 was determined after 31 days of start of systemic treatment (*N* = 27) were excluded to prevent an immortal time bias

^b^Hospitals are categorized in quartiles based on the hospital volume of all gastroesophageal cancer patients treated with systemic therapy in 2015 and 2016

### Treatment with trastuzumab

Details of systemic treatment and TTF were known in 1552 patients diagnosed in 2010–2015. HER2 was determined in 53% of these patients, of whom 17% were HER2 positive and 69% negative, and in 14% HER2 was unknown. Of the 141 HER2 positive patients, 77% (*n* = 108) received a trastuzumab-containing regimen, which increased from 60% (*n* = 35) in 2010–2013 to 88% (*n* = 73) in 2014–2015. OS increased in this period from 6.9 (IQR, 3.2–11.7) to 7.2 (IQR, 3.4–12.8) months (*P* = 0.079). Ninety-seven of trastuzumab-treated patients received it in first-line, and 11 patients beyond first-line treatment. In trastuzumab-containing regimens, chemotherapy backbones were doublets in 59%, triplets in 20%, and monotherapy in 11%. Most frequently used backbones were capecitabine/5-FU with oxaliplatin (*n* = 33) or with cisplatin (*n* = 31).

In HER2 positive patients, median TTF of first-line trastuzumab-containing therapy was 6.5 (IQR, 3.0, 11.7) and OS 11.6 (IQR, 5.3, 21.6) months, while TTF of nontrastuzumab-containing first-line treatment was 5.4 (IQR, 3.1, 7.4) and OS 6.6 (IQR, 5.1, 11.0) months (n = 33). In HER2 negative patients, median TTF of first-line treatment was 5.2 (IQR, 2.2, 9.0) and OS 7.5 (IQR, 3.9, 13.1) months.

## Discussion

Adequate HER2 testing is crucial for optimal decision-making on systemic treatment in metastatic gastroesophageal adenocarcinoma patients. In international guidelines it is therefore recommended to perform HER2 testing in all of these patients [8-10, 33]. In this nationwide cohort of 2846 patients with synchronous metastases and treated with palliative systemic therapy, HER2 testing increased over the study period from one in five to almost all patients. We found a large variety in the percentage of HER2 tested patients between the hospitals, and the volume of treated patients in a hospital independently associated with the probability of being tested for HER2.

Noteworthy, even in patients for whom the decision to be treated with systemic therapy had already been taken as is the case in our cohort still more than 10% of patients were not tested for HER2 in 2016. Even when we restrict our analysis to patients included in the ToGA study, i.e., with gastric or GEJ tumors, we still observed that 17% of gastric and 7% of GEJ adenocarcinoma patients diagnosed in 2016 were not tested. Male sex and treatment in lower hospital volumes were associated with a lower probability of HER2 assessment. Possible reasons for not testing could include contraindications for treatment with trastuzumab, and unawareness among physicians.

The HER2 overexpression rate of 19% is comparable with other studies [14-16]. In our study, this rate increased over time, probably because of the rise in tested esophageal adenocarcinomas, with a higher HER2 positivity rate compared to GEJ/cardia and stomach tumors. Overall, 23% of HER2 positive patients did not receive trastuzumab despite treatment with systemic therapy, which is remarkable as the additive side effects of trastuzumab are mild, while survival benefit is significant [6]. It cannot be excluded that financial reasons played a role [36]. For example, the reimbursement of trastuzumab could have been an issue if patients were not eligible for treatment with cisplatin, capecitabine or 5-FU, since the costs of trastuzumab are only covered when combined with this chemotherapy [37].

Furthermore, we found an interhospital variation in the proportion of HER2 tested patients of 29–100%, with a lower probability of undergoing HER2 assessment in low-volume compared to high-volume hospitals. A similar association with hospital volume was recently found in the probability of undergoing surgical treatment for gastric cancer [20]. Although we did not find a statistically significant association between hospital volume quartiles and, this was possibly a result of the limited number of patients in the lower hospital volumes. We did find this association when hospitals were categorized in two volume categories, which is in line with earlier published nationwide results [22]. This suggests HER2 assessment could increase if physicians of high-volume centers are involved in treatment decision-making, e.g., through regional multidisciplinary tumor boards.

Importantly, we found that in 26% of the HER2 assessments performed, ISH or MLPA was used despite a non-equivocal IHC result [28]. Reasons for additional ISH testing could include inadequate assessment of IHC staining due to HER2 heterogeneity or discordance between the primary tumor and metastasis [28, 38-40]. HER2 should therefore ideally be assessed on multiple specimens of the primary tumor, as well as on metastases, since HER2 targeted therapy is indicated if in one of the tumor specimens HER2 overexpression is observed [13].

HER2 positive patients treated with a trastuzumab-containing regimen had a longer survival compared to chemotherapy alone (11.6 and 6.6 months, respectively). However, survival in both groups was remarkably lower than in the ToGA trial (13.8 and 11.0 months, respectively) [6], probably due to restrictions in trial inclusion (e.g., performance status > 2), and because median age of our cohort was higher (64 versus 59 years in the ToGA trial). Nevertheless, the rise in trastuzumab administration over time could have contributed to the increased survival in our cohort from 6.9 (2010–2013) to 7.9 months (2014–2016).

Both HER2 positive and negative patients showed prolonged survival compared to non-tested patients. This supports the assumption of a selection of prognostically favorable patients that are tested for HER2, also endorsed by the higher number of tested patients without comorbidities compared to non-tested patients. Another explanation could be that non-tested patients are treated more frequently in low-volume hospitals. Moreover, the non-tested, but HER2 positive patients that did not receive trastuzumab could also have contributed to the lower survival in this group, since HER2 overexpression without targeted treatment is regarded a negative prognostic factor, although this is still subject of debate [41-43].

This is the first study in which real-world HER2 testing and outcomes of a nationwide gastroesophageal cancer cohort are described. However, the assumption that HER2 was not tested if it was not disclosed in pathology reports could have resulted in an underestimation of HER2 tested patients. Another limitation is the lack of information on reasons why HER2 testing was not performed or why trastuzumab was not administered. Lastly, as in any retrospective study, there were some missing data, which possibly hampered correction for confounding in multivariable analyses.

Our finding that still more than 10% of the patients treated with systemic therapy were not tested for HER2 is worrisome, not only because trastuzumab is currently the only targeted therapy in first-line palliative systemic treatment that has shown to improve survival rates, but also because other promising targets and biomarkers are on their way [44], such as programmed death-ligand 1 (PD-L1) [45, 46], Epstein-Barr Virus (EBV) [47, 48] and microsatellite instability (MSI) [49, 50] as biomarkers for checkpoint inhibition and selection in case of promising targeted therapies. Increased uptake of biomarker testing is therefore highly warranted in clinical practice.

In conclusion, daily practice management of metastatic gastroesophageal cancer has changed due to increased determination of HER2 status and administration of trastuzumab, which may have contributed to the improved survival in these patients over time. Advances in clinical practice could include a further increase in awareness of HER2 testing, especially in low-volume hospitals, and in trastuzumab administration.

## Electronic supplementary material

Below is the link to the electronic supplementary material.

Supplementary file1 (DOCX 20 kb)
