# Supplementary material for: Increased assessment of HER2 in metastatic gastroesophageal cancer patients: a nationwide population-based cohort study
Source: Gastric Cancer. 2020 Jan 11;23(4):579–90. doi: 10.1007/s10120-020-01039-7 (PMC7305095; doi:10.1007/s10120-020-01039-7)
Supplement: Supplementary file 1 — Supplementary file1 (DOCX 20 kb) [file 10120_2020_1039_MOESM1_ESM.docx]

**Supplementary Table 1. Baseline characteristics of patients in whom HER2 was tested (n=1524)**

|  | **HER2 negative (n=1035)**  **No. (%)** | **HER2 positive (n=285)**  **No. (%)** | **HER2 unknown (n=204)**  **No. (%)** |
| --- | --- | --- | --- |
| **ale** | 748 (72.3%) | 225 (78.9%) | 156 (76.5%) |
| **Age (years)** - median (IQR) | 63.0 (55.0, 69.0) | 63.0 (55.0, 69.0) | 63.0 (55.5, 70.0) |
| <50 | 398 (38.5%) | 107 (37.5%) | 75 (36.8%) |
| 50-64 | 381 (36.8%) | 113 (39.6%) | 75 (36.8%) |
| 65-79 | 238 (23.0%) | 61 (21.4%) | 50 (24.5%) |
| ≥80 | 18 (1.7%) | 4 (1.4%) | 4 (2.0%) |
| **Comorbidities** |  |  |  |
| 0 | 301 (29.1%) | 77 (27.0%) | 76 (37.3%) |
| 1 | 209 (20.2%) | 67 (23.5%) | 34 (16.7%) |
| ≥2 | 238 (23.0%) | 76 (26.7%) | 37 (18.1%) |
| Unknown | 287 (27.7%) | 65 (22.8%) | 57 (27.9%) |
| **Tumor location** |  |  |  |
| Esophageal | 339 (32.8%) | 159 (55.8%) | 72 (35.3%) |
| Gastroesophageal junction/cardia | 228 (22.0%) | 53 (18.6%) | 45 (22.1%) |
| Stomach (non-cardia) | 468 (45.2%) | 73 (25.6%) | 87 (42.6%) |
| **Tumor histology** |  |  |  |
| Adenocarcinoma NOS | 131 (12.7%) | 43 (15.1%) | 28 (13.7%) |
| Intestinal type adenocarcinoma | 493 (47.6%) | 194 (68.1%) | 109 (53.4%) |
| Diffuse type adenocarcinoma | 351 (33.9%) | 35 (12.3%) | 59 (28.9%) |
| Indeterminate type adenocarcinoma | 60 (5.8%) | 13 (4.6%) | 8 (3.9%) |
| **Tumor differentiation** |  |  |  |
| Well differentiated | 12 (1.2%) | 6 (2.1%) | 4 (2.0%) |
| Moderately differentiated | 169 (16.3%) | 73 (25.6%) | 32 (15.7%) |
| Poorly differentiated | 629 (60.8%) | 108 (37.9%) | 111 (54.4%) |
| Unknown | 225 (21.7%) | 98 (34.4%) | 57 (27.9%) |
| **Metastatic sites** |  |  |  |
| 1 | 589 (56.9%) | 132 (46.3%) | 93 (45.6%) |
| ≥2 | 446 (43.1%) | 153 (53.7%) | 111 (54.4%) |
| **Year of diagnosis** |  |  |  |
| 2010 | 51 (4.9%) | 7 (2.5%) | 15 (7.4%) |
| 2011 | 90 (8.7%) | 15 (5.3%) | 16 (7.8%) |
| 2012 | 123 (11.9%) | 31 (10.9%) | 29 (14.2%) |
| 2013 | 158 (15.3%) | 44 (15.4%) | 36 (17.6%) |
| 2014 | 199 (19.2%) | 67 (23.5%) | 29 (14.2%) |
| 2015 | 242 (23.4%) | 47 (16.5%) | 47 (23.0%) |
| 2016 | 172 (16.6%) | 74 (26.0%) | 32 (15.7%) |

*Tumor histology and differentiation are based on the primary tumor. Diffuse type tumors were classified as poorly differentiated. Abbreviations: IQR, interquartile range; NOS, not otherwise specified.*

**Supplementary Table 2. HER2 testing methods and results**

| **Reported IHC result** | **ISH/MLPA performed** | **Reported HER2 result** | **HER2 tests* (n=1764), No. (%)** | **Clinically unnecessary ISH/MLPA** | **Interpreted HER2 result** | **HER2 tested patients^#^**  **(n=1524), No. (%)** |
| --- | --- | --- | --- | --- | --- | --- |
| 0 | No | Negative | 451 (25.6%) |  | Negative | 498 (32.7%) |
|  | Yes | Negative | 141 (8.0%) | X |  |  |
| 1+ | No | Negative | 214 (12.1%) |  | Negative | 299 (19.6%) |
|  | Yes | Negative | 128 (7.3%) | X |  |  |
|  | Yes | Positive | 2 (0.1%) | X |  |  |
| 2+ | Yes | Negative | 269 (15.2%) |  | Negative | 238 (15.6%) |
|  | Yes | Positive | 80 (4.5%) |  | Positive | 73 (4.8%) |
|  | Yes | Unknown | 6 (0.2%) |  | Unknown | 3 (0.2%) |
|  | No | Negative | 1 (0.1%) |  | Unknown | 5 (0.3%) |
|  | No | Positive | 2 (0.1%) |  |  |  |
|  | No | Unknown | 8 (0.3%) |  |  |  |
| 3+ | No | Negative | 1 (0.1%) |  | Positive | 212 (13.9%) |
|  | No | Positive | 107 (6.1%) |  |  |  |
|  | Yes | Negative | 2 (0.1%) | X |  |  |
|  | Yes | Positive | 125 (7.1%) | X |  |  |
| Unknown | No | Negative | 107 (6.1%) |  | Unknown | 196 (12.9%) |
|  | No | Positive | 23 (1.3%) |  |  |  |
|  | Yes | Negative | 74 (4.2%) |  |  |  |
|  | Yes | Positive | 23 (1.3%) |  |  |  |

*IHC results that were reported to be 0-1+ or 1+ were scored as 1+, and 0-2+, 1-2+, 2+, or 2-3+ as 2+.*

**All HER2 tests that were performed. HER2 assessments that were performed as part of a study were not included, since these often include both IHC and ISH, regardless of the IHC result.*

*^#^If HER2 was tested multiple times, the last test result that was performed with a maximum of 31 days after start of systemic treatment is displayed. Abbreviations: IHC, immunohistochemistry; ISH, in situ hybridization; MLPA, multiplex ligation-dependent probe amplification.*
